# Prevalence and risk factors associated with amphistome parasites in cattle in Iran

**DOI:** 10.1002/vms3.330

**Published:** 2020-08-07

**Authors:** Nasser Hajipour, Fereshteh Mirshekar, Abolfazl Hajibemani, Mohammadreza Ghorani

**Affiliations:** ^1^ Department of Pathobiology Faculty of Veterinary Medicine University of Tabriz Tabriz Iran; ^2^ Young Researchers and Elite Club Zabol Branch Islamic Azad University Zabol Iran; ^3^ Department of Clinical Sciences Faculty of Veterinary Medicine University of Tabriz Tabriz Iran

**Keywords:** amphistomum, cattle, epidemiology, Iran, risk factor

## Abstract

Amphistomiasis, a neglected trematode infection of ruminants, has recently come up as an important reason for economic losses. The aim of this study was to determine the prevalence of bovine amphistomiasis and associated risk factors such as: age, gender, breed, season, water source, pastureland and grazing system. Between January 2016 and 2017, a total of 1,000 faecal samples and 1,000 rumens of cattle were collected from slaughterhouse of Zabol, Iran ante‐mortem and post‐mortem, respectively, and examined. The overall prevalence was 34.6% and 19.5% in terms of amphistome adults and eggs respectively. The identified amphistome species and their prevalence were *Paramphistomumcervi* (13.3%), *Cotylophoroncotylophorum* (19.5%), *Gastrothylaxcrumenifer* (5.9%) and *Carmyeriusspatiosus* (2.7%). The correlation between prevalence and season, age, breed, water source, pastureland and grazing system was significant (*p* < .0001). The presented information about the prevalence of amphistomes of cattle and individual and management risk factors can be used to design appropriate control measures.

## Graphical abstract

A significant correlation between amphistomosis infection and season, age, breed, water sour, pasture land and grazing system (*p* < .0001). Infection of the amphistomes parasite is a major problem for the cattle farmers of southeast of Iran. Amphistomiasis, a neglected trematode infectious of ruminants

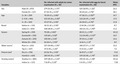

## 1 INTRODUCTION

Amphistomosis, a neglected trematode infection of ruminants, is caused by digena flukes belonging to several genera including: *Paramphistomum, Calicophoron, Cotylophoron, Explanatum, Gigantocotyle* and *Carmyerius*. Unfortunately, amphistomiasis has recently emerged as an important cause of productivity losses including decrease in milk and meat production, low nutrient conversion, weight loss and reduction in fertility (Javed Khan, Tanveer, Maqbool, & Masood, 2008; Mogdy et al., 2009; Soulsby, 1982). Adult amphistomes except *Explanatum explanatum*, which resides in the bile duct of domestic ruminants, can be found in and are the primary parasite of the rumen and reticulum of sheep, goats, cattle and water buffaloes (Mazahery, Razmyar, & Hoghooghi‐Rad, 1994). Whereas mild infection results in limited pathology, severe infections with massive number of immature parasites migrating through the intestinal tract cause acute parasitic gastroenteritis with high morbidity and mortality rates, particularly in young animals (González‐Warleta et al., 2013; Huson, Oliver, & Robinson, 2017; Zintl et al., 2014). Amphistomes have a heteroxenous life cycle with freshwater snails (*Bulinus truncatus*, *Planorbis planorbis*, *Indoplanorbis exustus, Bithynia bodiella* and *B. tentaculata*) serving as the intermediate hosts (Arfaa & Sahba, 1973; Sey & Eslami, 1981). Miracidia infect the mollusk with cercariae emerging and typically encysting on vegetation, hard surfaces or water becoming metacercariae that grazing ruminants (final host) ingest (González‐Warleta et al., 2013; Waal, 2010). The first location of the juvenile trematodes in the ruminant host is the small intestine where the parasites feed on the intestinal mucosa. Following further growth, the parasites migrate upwards to the reticulum and rumen where they spend the rest of their adult lives, shedding eggs that contaminate snail habitats (Ali et al., 2018; Dinnik & Dinnik, 1954; Soulsby, 1982). Amphistomosis is globally distributed, but the highest prevalence has been reported from tropical and sub‐tropical regions, particularly Africa, Asia, Australia, Eastern Europe and Russia (Dube & Aisien, 2010; Gupta, Singh, & Dutt, 1978; Horak, 1971; Huson et al., 2017). In Asia, *Paramphistomum cervi*, *P. explanatum*, *Gastrothylax crumenifer, Cotylophoron cotylophorum*, *Fischoederius elongates* and *Fischoederius cobboldi* have been recorded in India, Ceylon and China (Hanna, Williamson, Mattison, & Nizami, 1988; Wang et al., 2006). *Explanatum explanatum*, *Calicophoron calicophorn*, *Gastrothylax crumenifer*, *G. compressus*, *P. gotoi*, *P. cevri*, *P. microbothrium*, *P. gracile*, *Fischoederius* spp. and *Carmyerius spatiosus* were reported from Iran (Arfaa, 1962; Coskun, Eslami, Halajian, & Nikpey, 2012; Eslami, Halajian, & Bokaie, 2011; Khedri, Radfar, Borji, & Mirzaei, 2015; Mazahery et al., 1994; Nikpay, Houshmand, Eslami, & Bokaie, 2019; Otto & Eslami, 1980; Tehrani et al., 2015). The epidemiology of amphistomum parasites is determined by several factors governed by parasite–host–environment interactions. The major epidemiological variable influencing trematode burdens of animals is the infection rate from pastures. It is also influenced by the climatic requirement for egg hatching, development and survival of the miracidia and cerceria in pasture (Sintayehu & Mekonnen, 2012). Furthermore, the species of definitive and intermediate hosts, management system and grazing habits of the cattle (Horak, 1971), the biological potential and topography of the snail hosts (Horak, 1971; Swart & Reinecke, 1962a, b), the potential of the flukes to infect intermediate and definitive hosts (Dinnik & Dinnik, 1954) and finally climate impact the epidemiology and seasonal pattern of infection (Rolfe, Boray, Nichols, & Collins, 1991). In Iran, little information is available on the epidemiology and transmission dynamics of amphistomes. Although different studies have been conducted on Iranian freshwater snails, which serve as the intermediate hosts of parasitic trematodes including *Fasciola hepatica*, *Fasciola gigantica* and *Ornithobilharzia turkestanicum* (Imani‐Baran, Yakhchali, Malekzadeh Viayeh, & Paktarmani, 2012; Imani‐Baran, Yakhchali, MalekzadehViayeh, & Farahnak, 2013; Yakhchali, Mirrajei, & Malekzadeh‐Viayeh, 2013), the information about the intermediate hosts of amphistomes is not available.

The only relevant studies have concentrated on identification of species and prevalence of the amphistomes in ruminants (Arfaa, 1962; Eslami et al., 2011; Mazahery et al., 1994; Otto & Eslami, 1980; Tehrani et al., 2015). The objectives of this study were to estimate the prevalence of bovine amphistomiasis in southeast of Iran and investigate the potential risk factors associated with the disease.

## 2 MATERIAL AND METHODS

### 2.1 Study Area

This study was conducted in Zabol area in Sistan and Baluchestan province located in the south‐eastern part of Iran. This city is located in an area of 1,760 km^2^ laid on the border with both Sistan and Baluchestan. The study area receives 20–50 mm of rain annually and has a temperature ranging from −8 to + 48ºC. During the study period, it received an average of 3.25 mm rain and had temperature ranging from 10.7 to 36.5ºC (Table 1) (Organization, 2019).

**TABLE 1:** The mean temperature and precipitation in Zabol by month, 2016

Season	Winter			Spring			Summer			Autumn		
Month	J	F	Mar	A	Ma	Ju	Jul	Au	S	O	*N*	D
Precipitation (mm)	7.7	0	22.1	1.7	7	0	0	0	0	0	0.1	0.4
Temperature (°C)	10.7	13.1	19.4	24.1	31.8	34.2	36.5	33.7	31.9	21.9	13.4	11.8

Abbreviations: J, January; F, February; Mar, March; A, April; Ma, May; Ju, June; Jul, July; Au, August; S, September; O, October; *N*, November; D, December.

### 2.2 Sampling

From January 2016 to January 2017, 1,000 cattle were randomly selected in four seasons at the abattoir of Zabol, southeast of Iran. In the ante‐mortem examination, potential risk factors such as breed (native and Indian), age (<2, 2–4 and > 4 years old), sex (male and female) and management risk factors including grazing type (indoor, outdoor), water source (tap, river) and pasture land (humid and dry) were recorded. Age of the animals was estimated using the eruption of permanent incisor teeth criterion as described by Curasson (1947). Management risk factors were obtained through interviewing with owners of the animals and recording in previously prepared questionnaires. In the abattoir, the cattle were examined 4 days per week, 16 times per month, throughout the year. During the study period, approximately 10 g of faecal samples were collected directly from the rectum of the animal or from the top of freshly defecated uncontaminated faeces in a clean plastic container after labelling with specific identification number, transported to the laboratory and stored at 4°C until the test was performed within 48 hr. During post‐mortem examination, the rumen and reticulum were systematically inspected for the presence or absence of adult amphistomes using the routine meat inspection procedures (Ayalew, Tilahun, Aylate, Teshale, & Getachew, 2016; Szmidt‐Adjidé et al., 2000). If evidence of adult amphistomes was found, it was recorded separately and stained using aceto‐alum carmine.

To determine prevalence of forage contamination, 10 commonly used grazing areas in the region were divided into five sections (approximately 500 square meters) and 200 forage samples were collected from each section. A total of 1,000 samples were collected and included fenugreek (*Trigonella foenum‐graceum L*.) (*n* = 478), alfalfa (*Medicago sativa L*.) (*n* = 322), Red Clover (*Trifolium pretense*) (*n* = 100) and Chay‐Oti (*Chamaesphacos ilicifolius*) (*n* = 100). Samples were transported to the laboratory for analysis in sterile nylon bags. Fresh forage (*n* = 735) immediately were tested and a number of sample (*n* = 265) were dried in the sun and used as dry forage.

### 2.3 Parasitological techniques

Faecal samples were examined by sedimentation technique for the presence of fluke eggs using the method described by Adejoju, Bamidele, and Olakunle (2008). The technique was performed with 10 g of faeces to which 200 ml of water was added and mixed. The mixture was filtered three times through a sieve with pore size 0.25 mm. The filtrate was allowed to stand for 10 min after which the sediment was collected in a test tube and centrifuged at 1,000 g for 3 min. After centrifugation, the supernatant was decanted and a drop of the sediment was tested microscopically. Amphistomum eggs were identified based on morphology (Soulsby, 1982). Flukes recovered from each infected animal during the post‐mortem examination were counted and morphologically identified as described by Soulsby (1982); Urquhart, Armour, Duncan, Dunn, and Jennings (1996). Each fresh and dry forage sample was weighed (100 g) into sterile plastic bags and washed with physiological saline solution (0.85% NaCl) and the washing water/saline was left for about 24 hr for sedimentation to take place. The top water was discarded and 5 ml of the remaining washing water centrifuged at 2000 g for 5 min. The supernatant was discarded and the residue carefully collected. The samples were agitated gently by hand in a physiological saline solution containing lugol's iodine and then were examined through light microscopy (Adanir & Tasci, 2013).

### 2.4 Data analysis

Correlation between the infection rate and individual and management risk factors was assessed using the Chi‐square test. The data were analysed using SPSS software version 21 (Chicago, IL, USA) and *p* < .001 or *p* < .01 was considered as significant.

## 3 RESULTS

Of the total 1,000 rumens inspected for determining the prevalence of adult amphistomes, 346 (34.6%) animals were positive (Table 2). The identified species of adult amphistomes and their prevalence were *Paramphistomum cervi* (13.3%), *Cotylophoron cotylophorum* (19.5%), *Gastrothylax crumenifer* (5.9%) and *Carmyerius spatiosus* (2.7%). The overall prevalence of cattle paramphistomiasis examined by faecal sedimentation test was 19.5% (195 samples with eggs of 1,000 faecal samples). The contamination rate of fresh forages with metacercariae and eggs of amphistomum was significantly higher (58.77%) than those of dry forages (33.96%). Based on the analysed data, the correlation between the prevalence of amphistomum in the animals and management and individual risk factors was significant (Table 2). Significantly higher prevalence was found in the female compared with the male cattle, in summer than the other seasons, for Indian breed than native breed, in humid over the dry pasture land, in free grazing than the indoor systems and in river than tap water source (*p* < .0001).

**TABLE 2:** Prevalence of infection with amphistome parasites by different epidemiological aspects

Variables		No. infected with adults by rumen examination (% ± SE)*	No. infected with eggs by faecal examination (% ± SE)*	Mean EPG
Sex	Male (*n* = 873)	279 (31.96 ± 1.57)^a^	165 (18.90 ± 1.32)^a^	16.2
	Female (*n* = 127)	67 (52.76 ± 4.44)^b^	30 (23.62 ± 3.78)^a^	18.1
Age	2> (*n* = 259)	78 (30.12 ± 2.80)^a^	47 (18.15 ± 2.30)^ab^	14.7
	2–4 (*n* = 496)	225 (45.36 ± 2.20)^b^	122 (24.59 ± 1.90)^a^	17.3
	4< (*n* = 245)	43 (17.55 ± 2.40)^a^	26 (10.61 ± 6.70)^b^	11.8
Breed	Native (*n* = 155)	18 (11.62 ± 2.50)^a^	13 (8.38 ± 2.0.20)^a^	8.7
	Indian (*n* = 845)	328 (38.81 ± 1.67)^b^	182 (21.53 ± 1.40)^b^	11.6
Season	Spring (*n* = 250)	70 (28 ± 2.84)^a^	28 (11.2 ± 1.99)^a^	10.12
	Summer(*n* = 250)	150 (60 ± 3.10)^b^	112 (44.80 ± 3.15)^bc^	16.9
	Autumn (*n* = 250)	100 (40 ± 3.10)^c^	39 (15.6 ± 2.30)^c^	13.6
	Winter (*n* = 250)	26 (10.4 ± 1.93)^d^	16 (6.4 ± 1.5)^d^	8.9
Water source*	River (*n* = 653)	327 (50.08 ± 1.95)^a^	180 (27.57 ± 1.75)^a^	13.2
	Tap (*n* = 347)	19 (5.48 ± 1.22)^b^	15 (4.32 ± 1.09)^b^	7.2
Pasture land*	Dry (*n* = 376)	63 (16.76 ± 1.92)^a^	36 (9.57 ± 1.51)^a^	9.12
	Humid (*n* = 624)	283 (45.35 ± 1.99)^b^	159 (25.48 ± 1.74)^b^	13.23
Grazing system*	Outdoor (*n* = 885)	339 (38.31 ± 1.63)^a^	190 (21.46 ± 1.38)^a^	10.12
	Indoor (*n* = 115)	7 (6.09 ± 2.23)^b^	5 (4.35 ± 1.91)^b^	7.28

* Values with different superscripts along the same column are different significantly (*p* < .0001).

## 4 DISCUSSION

Being aware of risk factors associated with amphistomiasis in cattle is an important pre‐requisite for the design and implementation of effective control strategies and management programmes that can lead to the control and eradication of the disease. In addition, it is a good aid for clinical diagnosis, determination of epidemiology and patterns of the disease. The present study revealed that the overall prevalence of amphistomidae flukes at Zabol, southeast of Iran was 34.6% during post‐mortem examination of rumen and reticulum for presence of adult amphistomum and 19.5% during ante‐mortem faecal inspection for presence of amphistomum eggs. From the perspective of the adult flukes, our results were in agreement with previous studies in which the infection rate with paramphistomidae flukes was as reported 33.9% in Mazandaran, Northern Iran (Eslami et al., 2011) and 36.9% from South‐Eastern Iran (Khedri et al., 2015). In our research, the infection rate was higher than what was reported from Guilan province in northern Iran (19.70%) (Nikpay et al., 2019), in north‐western Iran (0.041%) (Tehrani et al., 2015), Algeria (12.1%) (Titi, Mekroud, Sedraoui, Vignoles, & Rondelaud, 2010), Spain (18.8%) (González‐Warleta et al., 2013) and Turkey (8.95%) (Ozdal, Gul, Ilhan, & Deger, 2010). The results obtained from the faecal sedimentation method in the present study (19.5%) was lower than the prevalence reported by Yeneneh, Kebede, Fentahun, and Chanie (2012) from northwest Ethiopia (45.83%) and Pfukenyi, Mukaratirwa, Pfukenyi, and Willingham (2005) from Zimbabwe (29.5%). However, the identified prevalence infection in this study was higher than that of the earlier research, conducted on central France (15%) (Mage, Bourgne, Toullieu, Rondelaud, & Dreyfuss, 2002). This inconsistency is probably due to differences in sample size, diagnostic technique, climatic conditions, ecological and management systems. In addition, given the quantity of sediment examined in the study presented here, the number of faeces with eggs might have been underestimated. All identified amphistomum species in this study have been recorded previously by Khedri et al. (2015) and Coskun et al. (2012). It should be noted that morphological identification can be challenging; therefore, molecular methods are required to confirm differences in prevalence of these species. Our studies revealed the prevalence of *C. cotylophorum* (19.5%), *P. cervi* (13.3%), *G. crumenifer* (5.9%) and *C. spatiosus* (2.7%) was lower than the estimated prevalence previously found in south‐eastern Iran by Nikpay et al. (2019) with prevalence of 20, 20, 40 and 15% respectively. The infection was non‐significantly higher in female cattle than that of male cattle, which was in agreement with the results obtained from Ethiopia (Ayalew et al., 2016; Yeneneh et al., 2012) and south‐eastern Iran (Khedri et al., 2015). According to Tariq, Chishti, Ahmad, and Shawl (2008), the higher rate of amphistomiasis in female than male cattle could be due to genetic predisposition and differential susceptibility owing to hormonal effects. Additionally, it could be attributed to stress stemming from pregnancy and lactation and insufficient feed supplements which are absolutely required for reproductive and productive activities. The aforementioned parameters can suppress immune status of females and thereby increase prevalence of amphistomes in case of females than their male counterparts (Bilbo & Nelson, 2001). In addition, this finding may be due to the higher mean age of females than males, with females being slaughtered at an older age (Hajipour & Tavassoli, 2019). There were statistically significant differences (*p* < .0001) between the prevalence of amphistomum and that of breed, age groups, season, water source, type of grazing area and grazing system of the animals. The result for Indian breed (38.81%) was significantly higher than native breed (11.62%) which was consistent with the study carried out by Khedri et al. (2015). They have suggested that native breed has more tolerance to parasitic diseases. The rate of infection in 2‐ to 4‐year‐old (45.36%) cattle was significantly higher than the others, which was similar to studies carried out by Nikpay et al. (2019), although they showed that there was no significant difference. This is probably due to the fact that amphistomiasis of cattle is largely a disease of young animals, as successive small infections produce an almost complete immunity (Vercruysse & De Bont, 2001). Significantly (*p* < .0001) higher infection of amphistomes was recorded during summer season (60%) followed by autumn (40%), spring (28%) and winter (10.4%). Our findings were in accordance with Bansal et al. (2018) in sheep from India and Javed Khan et al. (2008) in cattle from Pakistan. However, Nikpay et al. (2019), Ozdal et al. (2010) and Eslami et al. (2011) showed that the highest infection rate of amphistomum was in spring, autumn, summer and winter respectively.

According to the Table 1, the amount of rainfall and temperature in the season of spring in the area studied were suitable for developing miracidium in egg, hatching, growth and emerging of cercariae from snails (intermediate hosts) and this condition causes the number of metacercariae to increase in forage next to streams, then infective metacercariae may be swallowed by cattle and within a pre‐patent period of 2 months one can see an increase in amphistomiasis in summer (Soulsby, 1982). The rate of infection by amphistomum parasites was significant in cattle grazing in wetlands and drinking from river water in comparison with the animals that were grazing in dry lands and drinking from tap water. In addition, the rate of infection among cattle that had grazed in indoor areas was significantly lower in comparison with the cattle which had free grazing. These results were consistent with the results obtained in the study of Pfukenyi et al. (2005). Although the population and species of snails were not investigated in the present study, but it was observed that the dry land and tap water were not suitable environments for snail growth and metacercariae production (Dinnik, 1964). In indoor system, animals had not access to pastures and were fed on dry grass in which the rate of contamination with metacercariae were low and these factors can be considered among the reasons for observed decrease in infection among the studied animals.

The results of our studies showed that the contamination rate of fresh forages (58.77%) with eggs and metacercariae stage of amphistomum were higher than those in dry forages (26.41%). Probably the reason is that survival of eggs and metacercariae of amphistomum on dry forages is low and these may be lost due to the heat of sunlight during the storage period of the forage.

Due to the fact that snails control by managing habitats can be done by removing herbal barriers (which decreases the snails access to the food) and also by increasing the rate of water flow in rainy seasons (Horak, 1971; Woolhouse & Chandiwana, 1990) and due to the fact both methods are difficult in the area studied, attention to the grazing management factors can be somewhat effective in decreasing the infection rate in cattle.

## 5 CONCLUSION

Our findings are of great significance to elucidate different aspects of the epidemiology of the disease in these parts of Iran, where formulating a control program should be taken into consideration. In order to effectively control amphistomosis in this geographical region, further studies including infections caused by immature forms, identification of the species using molecular techniques and also study on intermediate host snails should be done. However, the results of this present study showed that use of tap water, dry pasture land, native breeds, dry grasses and closed grazing system can be considered as control strategy for amphistomiasis.

## CONFLICT OF INTEREST

The authors declare that they have no conflict of interest.

## AUTHOR'S CONTRIBUTIONS

Nasser Hajipour, Fereshteh Mirshekar, Mohammadreza Ghorani and Abolfazl Hajibemani made substantial contributions to the conception and design, acquisition of data, analysis and interpretation of data and drafting of the manuscript; Nasser Hajipour and Fereshteh Mirshekar participated in the sampling, diagnosis, technical supports and revised manuscript. Mohammadreza Ghorani and Abolfazl Hajibemani participated in the design, coordination of the study and data analysis revision. Nasser Hajipour assisted with drafting and final revising the manuscript. All authors read and approved the final manuscript.

## AUTHOR CONTRIBUTION

Nasser Hajipour: Conceptualization; Data curation; Funding acquisition; Investigation; Methodology; Project administration; Supervision; Validation; Visualization; Writing‐original draft; Writing‐review & editing. Fereshteh Mirshekar: Data curation; Investigation; Methodology. Abolfazl Hajibemani: Data curation; Formal analysis; Software; Visualization; Writing‐review & editing. Mohammadreza Ghorani: Formal analysis; Investigation; Resources; Software; Validation.

6

**TABLE 3:** The rate of contamination of fresh and dry forage with amphistomum eggs and the metacercariae

Type of forage	No. sample	No. contaminated (%)*	No. contaminated metacercariae (%)*	No. contaminated egg (%)*	No. contaminated mix (%)
Dry	265	70 (26.41)^a^	20 (7.54)^a^	50 (18.86)^a^	0
Fresh	735	432 (58.77)^b^	325 (44.21)^b^	150 (20.40)^b^	40 (9.19)
Total	1,000	502 (50.2)	345 (34.5)	200 (20)	40 (9.19)

* Values with different superscripts along the same column are different significantly

7

### PEER REVIEW

The peer review history for this article is available at https://publons.com/publon/10.1002/VMS3.330.
